# Urgency an important factor when assessing fecal incontinence

**DOI:** 10.1007/s13304-024-01975-4

**Published:** 2024-09-06

**Authors:** Louise Almkvist, Ulf Gunnarsson, Karin Strigård

**Affiliations:** Department of Diagnostics and Intervention, Surgery, Umeå University, SE-901 87, Umeå, Sweden

**Keywords:** Fecal incontinence, Scoring methods, Colorectal surgery, General surgery, Proctology

## Abstract

The aim of this study was to investigate if Low Anterior Resection Syndrome (LARS) score contributed with complementary information to Wexner score when assessing fecal incontinence (FI). The hypothesis was that LARS score would be likely to provide complementary information to Wexner score in the assessment of FI regardless of etiology. LARS score has been used as a complement to traditional scoring systems to assess bowel dysfunction, targeting FI among patients after radical cystectomy, in women with endometriosis, and in colorectal cancer patients. Wexner score as a single tool does not address the complexity of FI and urgency, a disabling symptom. A retrospective cohort study at a surgical outpatient clinic included patients diagnosed with FI who answered LARS and Wexner scores questionnaires at their first visit to the clinic between 1st January 2015 and 31st December 2018. Kendall’s tau, Spearman rank correlation, Cohen’s kappa, and scatterplots were analyzed for participants and specific subgroups to assess any correlation and agreement between answers to the two scoring systems. One hundred nineteen patients met the inclusion criteria, one hundred eight women and eleven men. Kendall’s tau ranged from 0.32 to 0.39, indicating lack of correlation. Correlation coefficients using Spearman rank ranged from 0.36 to 0.55, *i.e.,* only fair to moderate correlation. Kappa was 0.21–0.28, *i.e.*, only slight to fair agreement. Distribution of LARS and Wexner scores in the scatterplot showed wide variability and lack of agreement. Combined use of both the Wexner and LARS scores provided complimentary information, and thus a more complete mapping of FI as well as taking all entities in consideration.

## Introduction

Fecal incontinence (FI) is the involuntary leakage of solid or liquid stool [1]. Inadequate reservoir capacity, abnormal sphincter function, pelvic floor dysfunction, altered stool consistency, and inadequate rectal sensation may all result in FI [2, 3]. FI has a negative impact on a person’s everyday life [4–8], affecting a large part of the population [3, 9, 10] with a complex variety of symptoms.

Guidelines for diagnosis, treatment, and an evidence-based treatment algorithm for FI have been published by Assmann et al. [11]. In the assessment of bowel habits, scoring systems targeting FI and bowel dysfunction are valuable tools when mapping patient symptoms and evaluating treatment results. The multi-etiology of FI results in a variety of clinical pictures and manifestations, making its assessment complicated [12]. Categorization of FI depending on presence of passive leakage or urgency is of clinical relevance [13, 14].

There are several different scoring systems for the assessment of FI or anal incontinence, the Wexner score being the one most used by clinicians [15]. Wexner score is validated for assessment of FI regardless of etiology [2]. LARS score is used to assess bowel dysfunction after low anterior resection (LAR) for rectal cancer [16]. LARS score also provides valuable information when assessing bowel dysfunction after radical cystectomy, after surgery for endometriosis, and after surgery for colon cancer [17–20]. Normative data for LARS score have been published by Juul et al. Factors associated with major LARS in a general population were physical disease and female sex. Among females between 50 and 79 years, 19% reported major LARS, while 10% of males in the same age group presented with major LARS [21].

Wexner score alone in the evaluation of FI does not address the complexity of FI, since all disabling symptoms should be taken into consideration, e.g., urgency which is not addressed by Wexner score. LARS score targets bowel dysfunction and focuses on the important domains of urgency.

The authors’ hypothesis was that LARS score provides unique, valuable information, and complement Wexner score when assessing FI in general and, therefore, does not correlate fully with Wexner score.

The aim of this study performed at a colorectal outpatient clinic was to see if the Low Anterior Resection Syndrome (LARS) score provides complementary information to the Wexner score when assessing patients with FI, regardless of the underlying etiology.

## Methods

This retrospective cohort study was conducted and reported in accordance with the STROBE checklist for cohort studies [22].

Inclusion criteria were: 1. first visit to the surgical colorectal outpatient clinic at Umeå University Hospital between 1st January 2015 and 31st December 2018; 2. diagnosis of FI regardless of etiology according to the International Classification of Diseases-10-SE [23] code R15.9; and 3. both LARS and Wexner questionnaires answered at the time of the first outpatient visit. Both scoring systems were explained to the patient by a surgeon at the first visit.

Exclusion criteria were missing LARS and/or Wexner score data or no data from follow-up office visit to the colorectal outpatient clinic.

A database using Microsoft^®^ Access^®^ 2013 was set up. Potential participants were selected after reviewing medical records in which FI was registered under the diagnosis code R15.9.

Notes made by the surgeon in the patient’s medical record were used to determine the etiology of FI. In cases where information was limited or when no clear cause was indicated by the surgeon at the first outpatient visit, all diagnoses registered in the records were reviewed.

Date of birth, date of first visit to the surgical outpatient clinic, sex, body mass index (BMI) kg/m^2^, and comorbidities were registered. Results of anorectal manometry and 3D anal ultrasound including injuries observed and registered by location (internal anal sphincter (IAS), external anal sphincter (EAS), or the puborectal muscle) were noted. Radiological examination results (defecography or transit time), any intervention, etiology of FI, and LARS and Wexner scores were registered.

Graphical contents, figures, and statistical analyses were performed in STATA^®^ 13.1 (StataCorp 4905 Lakeway Drive Collage Station, Texas 77,845 USA) and were used to measure correlation and agreement between LARS and Wexner scores. Statistical methods were discussed with a senior statistician with expertise in the field of medicine.

Kendall’s tau was used to calculate correlation between LARS score when divided into categories (no LARS 0–20 points, minor LARS 21–29 points and major LARS 30–42 points) and total Wexner score. Spearman rank correlation was used to calculate correlation and agreement between overall LARS and Wexner scores. Both total LARS score, total Wexner score, and LARS score divided into its three existing categories were ordinal variables. Spearman rank correlation was used when analyzing total LARS and Wexner scores. When analyzing LARS categories and total Wexner score, Kendall’s tau was used because of the limited number of outcomes when analyzing LARS categories.

For assessment of agreement, square matrices measuring 3 × 3 and scatterplots were constructed. To enable statistical analyses, matrices were constructed using existing LARS score categories and by dividing Wexner score into three categories according to total score (1–5 points, 6–10 points and > 10 points). Cohen’s kappa was used to measure agreement.

Statistical tests were also used to calculate agreement and correlation after categorizing data into the subgroups ≥ 50 years of age, < 50 years of age, injury to IAS or EAS or both, and no observed injury to the anal sphincter.

Approval by the Regional Ethics Review Board in Umeå was granted in 2017 and in 2018, identification numbers 2017/189-31 M and 2018–217-32 M.

## Results

### Background information of the cohort

**Fig. 1:**
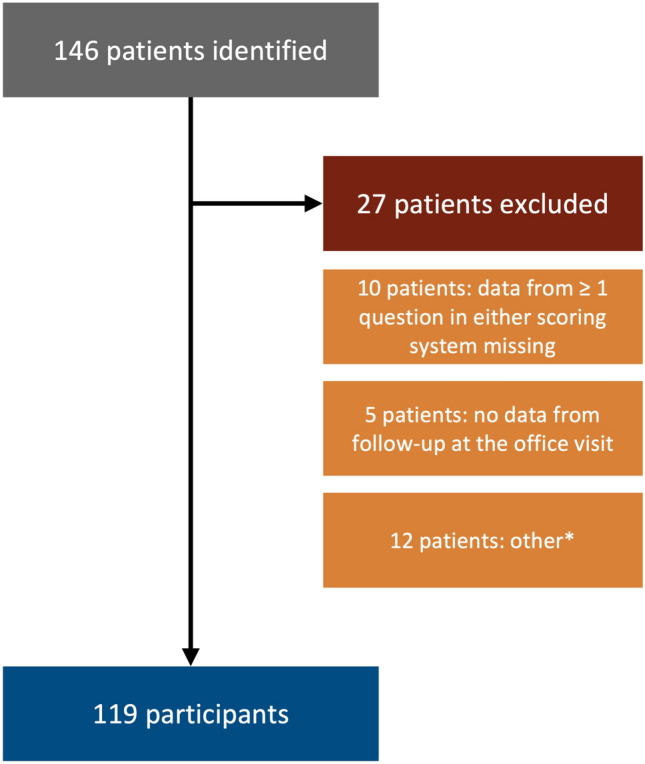
Flow chart. 119 of 146 met the inclusion criteria (twenty-seven patients excluded). Patients with no data from follow-up office visit to the colorectal outpatient clinic were excluded

A total of 119 out of 146 patients met the inclusion criteria and were included in the study. All participants had been examined and evaluated by a senior colorectal surgeon with expertise in coloproctology. Patients excluded and reasons for exclusion are presented in Fig. 1. Two other reasons for exclusion were that the first visit did not include evaluation and examination by a surgeon, e.g., visited a stoma care nurse for assessment and diagnosis, and that FI was not registered at the first outpatient visit.

**Fig. 2:**
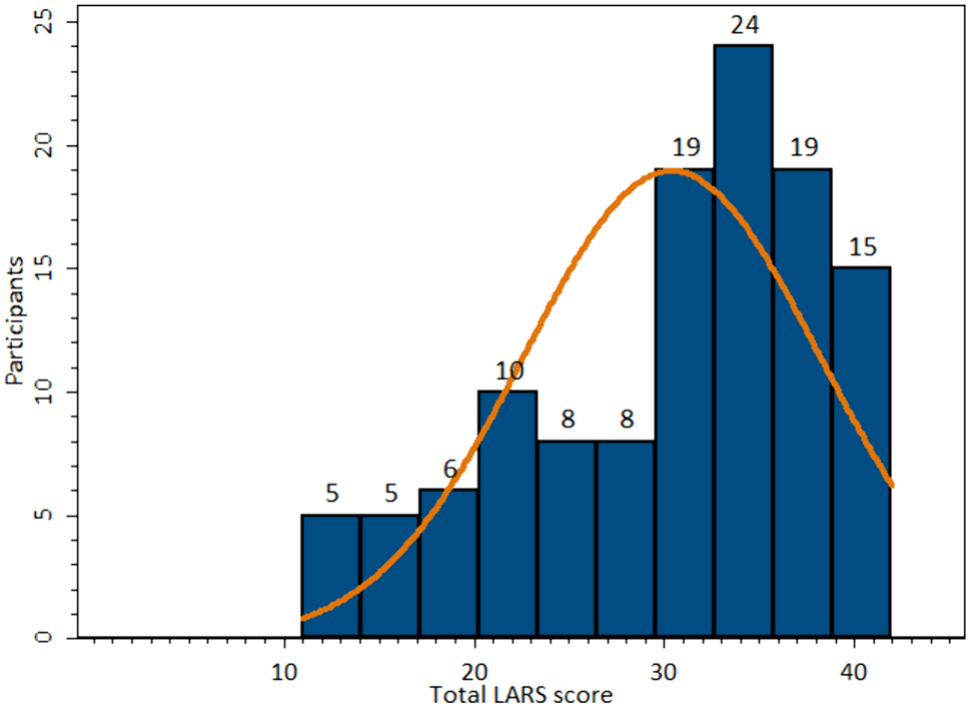
Histogram LARS score. The distribution of total LARS score between all participants

**Fig. 3:**
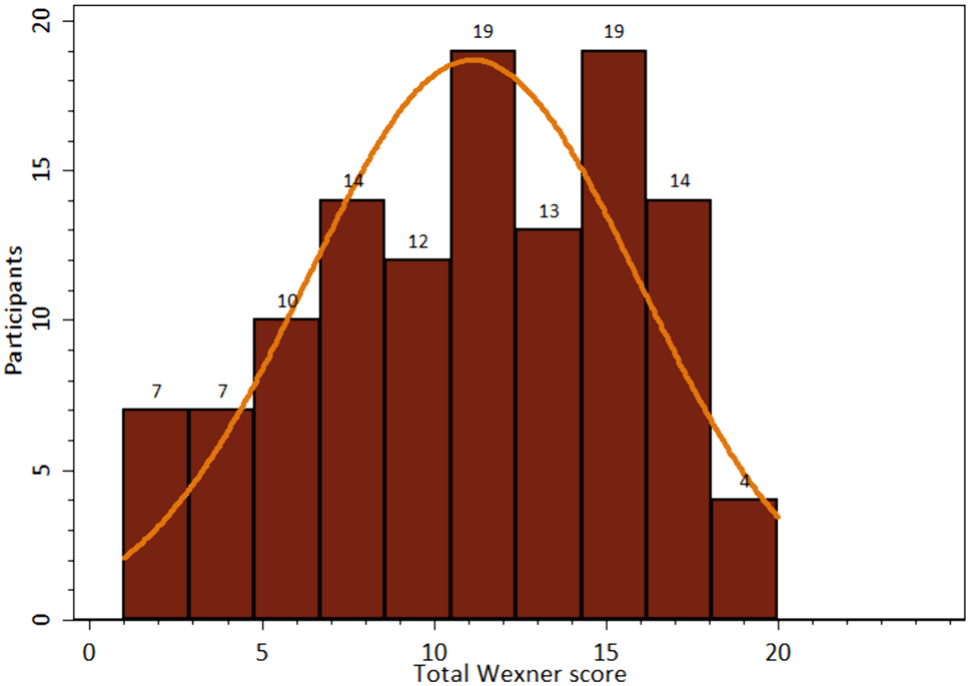
Histogram Wexner score. The distribution of total Wexner score between all participants

Distribution of total LARS and Wexner scores are presented in Figs. 2, 3. For participant characteristics, see Table 1. Of the 119 patients included, 108 were women and 11 men. Mean age of the 119 participants was 54 years of age (minimum 22 and maximum 88 years of age, SD 17.9) and mean BMI was 26.6 kg/m^2^ of 92 of the participants (minimum 18 and maximum 43 kg/m^2^, SD 4.9).

**Table 1:** Participants’ characteristics

Etiology		3D-endoanal ultrasound		Anorectal manometry I			Anorectal manometry II		
Obstetric injuries	66	Number of participants examined	116	Number of participants examined		22	Number of participants examined		58
Unknown/other	39	Injuries to IAS	10	Mean resting pressure (mmHg)	Min	8	Mean resting pressure (mmHg)	Min	− 38.8
					Max	65		Max	87.5
					Mean	35.6		Mean	26.0
Multiple etiologies	6	Injuries to EAS	18	Mean pressure increase voluntary tonus (mmHg)	Min	15	Maximal voluntary sphincter pressure (mmHg)	Min	7.7
					Max	146		Max	254.9
					Mean	47.1		Mean	44.0
Complication of surgical procedure	4	Injuries IAS and EAS	46	Normal examination		1	Normal examination		2
Radiation therapy	1	Injury to the puborectal muscle	1	Pathological examination		21	Pathological examination		56
Inflammatory bowel disease	1	Injuries to IAS, EAS and the puborectal muscle	2						
Neurological conditions	1	Normal examination	38						
Post gynecologic surgery	1	Data missing	1						

Characteristics of all participants included in the study. The majority (*n* = 108) of participants were women, only 11 participants were men. Different etiologies could be seen among the 119 participants. The most common cause of FI was obstetric injuries. Causes of FI in the presented cohort involved traumatic injuries to the pelvic floor, rectocele, high consumption of laxatives, previous ischemic stroke, disc herniation, previous transanal endoscopic microsurgery, sphincterotomy due to anal fissure, previous Low Anterior Resection, post colporrhaphy, post-surgery for anal fistula and hemorrhoids etc. 3D-endoanal ultrasound was performed by experienced colorectal surgeons. Results of anorectal manometry are presented in two different cohorts due to changes regarding examination protocol made by the Department of Clinical Physiology

Of the 119 participants, 116 had been examined with 3D anal ultrasound, 80 with anorectal manometry, 5 with defecography (2 showing pathology), and 1 had been referred for transit time radiography. Ten participants had an IAS injury alone, eighteen had an EAS injury alone, and one had an injury to the puborectal muscle. Forty-six had multiple injuries located at both the IAS and EAS, two participants had injuries to the IAS, EAS, and the puborectal muscle. Thirty-eight participants had no injury detected, and results were missing for one who had been examined with 3D anal ultrasound.

**Fig. 4:**
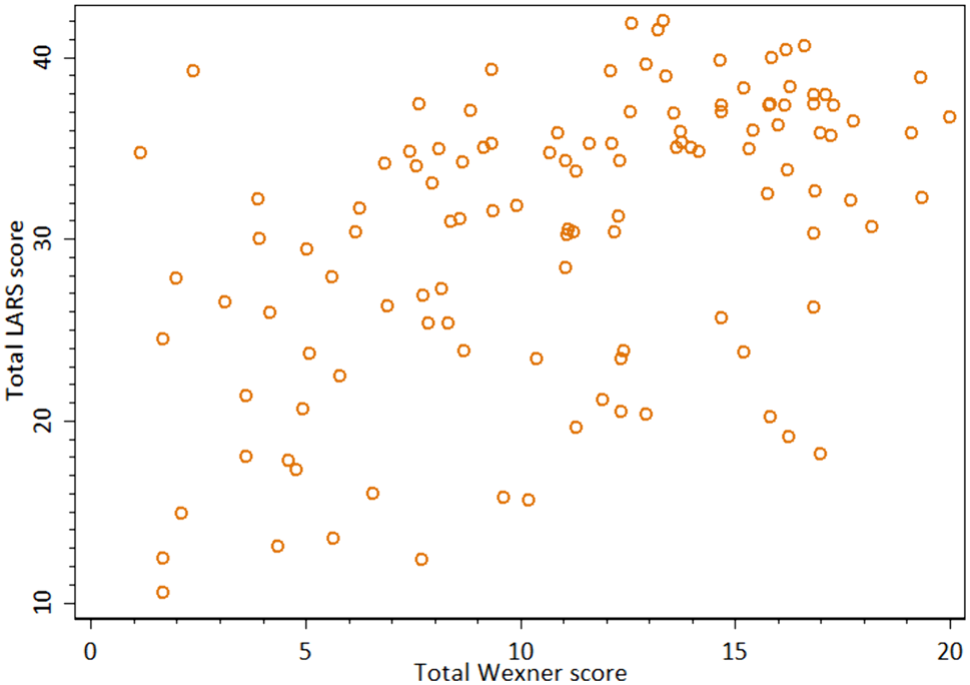
Scatterplot total LARS and Wexner scores. Scatterplot of distribution of total LARS and Wexner scores of all 119 participants showing great variability

The most common cause of FI was an obstetric injury (66 participants, Fig. 4). Other causes of FI were complication of surgical procedure including transanal endoscopic microsurgery, low anterior resection for rectal cancer, sphincterotomy, and fistulotomy of an anal fistula. Neurological conditions included spinal disc herniation affecting nerve roots L5-S1. The category unknown/other included excessive laxative treatment, lactose intolerance, irritable bowel syndrome, anal fissure, rectocele, traumatic injury due to sexual abuse, traumatic injury of other origin, and previous surgery for hemorrhoids. Six participants presented with a combination of causes.

The majority (*n* = 43) of participants had received several interventions to treat FI. Of those who had received 1 intervention, 19 participants received biofeedback therapy, 9 sphincteroplasty, 2 dextranomer and sodium hyaluronate injection, and 13 participants had sacral nerve stimulation. Ten participants had not received an intervention, and twelve some other form of intervention.

### Correlation analyses and Cohen’s kappa

Correlation results using Kendall’s tau and Spearman rank correlation are presented in Table 2. When estimating correlation using Spearman rank between total LARS and Wexner scores, there was some correlation in the subgroup *injury located at IAS, EAS or both* (0.55 compared to the other subgroups, Table 2). Kappa values showed slight to fair agreement (0.21–0.28, Table 2).

**Table 2:** Statistical analyses of LARS score and Wexner score

		All participants	< 50 years of age	≥ 50 years of age	No injury to the anal sphincters	Injury to IAS, EAS or both
N		119	48	71	39	76
Kendall’s tau	LARS score divided into categories and Wexner score	0.37	0.32	0.32	0.37	0.39
Spearman rank correlation	Total of LARS and Wexner scores	0.50	0.36	0.45	0.42	0.55
Cohen’s kappa	LARS score divided into categories and Wexner score divided into categories	0.28	0.21	0.24	0.26	0.28

To measure correlation and agreement between LARS and Wexner scores, Kendall’s tau and Spearman rank correlation, and Cohen’s kappa were used. Wexner score was divided into following categories; 1–5, 6–10, and > 10 points. Spearman rank correlation test, Kendall’s tau, and Cohen’s kappa show sign of poor agreement between the two scores

### Distribution of LARS and Wexner scores

**Fig. 5:**
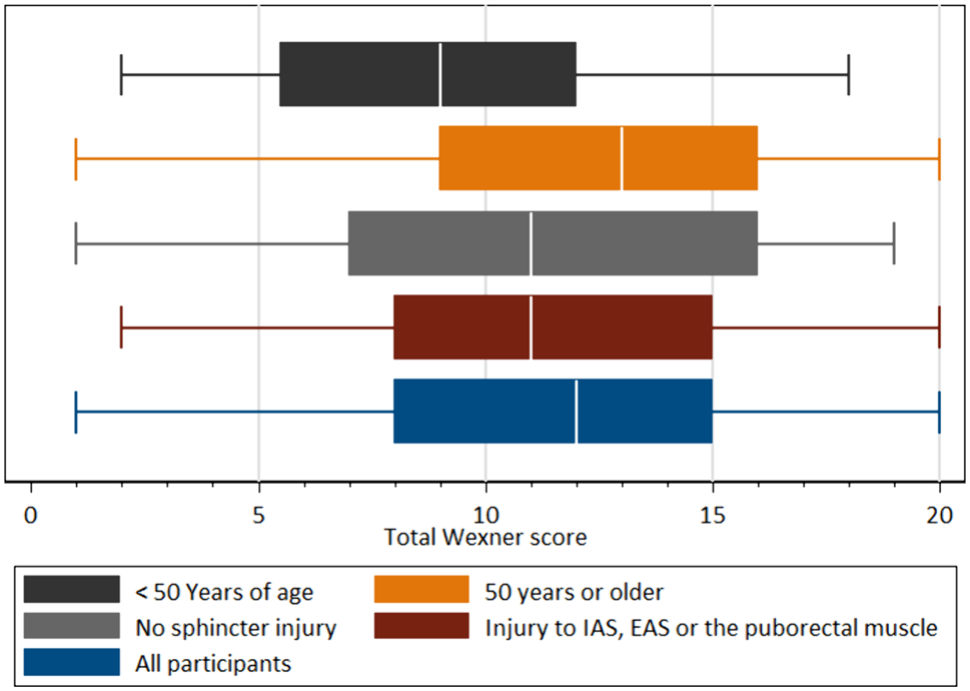
Box plot distribution Wexner score. Distribution of total Wexner score for all participants and for the different subgroups presenting minimum, maximum, and median for Wexner score

**Fig. 6:**
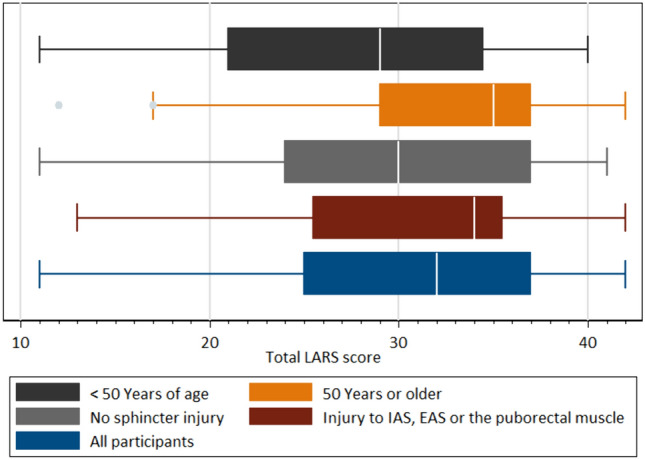
Box plot distribution LARS score. Distribution of total LARS score for all participants and for the different subgroups presenting minimum, maximum, and median for LARS score

Figure 4 shows the distribution between total LARS and Wexner scores on a scatterplot. Figures 5, 6 show distribution of total LARS and Wexner scores between participants and for each subgroup, presenting minimum, maximum, and median values.

## Discussion

LARS score provides valuable complementary information in addition to Wexner score when assessing FI as the results in this study show. The distributions of total LARS and Wexner scores presented in Fig. 4 show great variation, and statistical methods further illustrated this (see Table 2).

Scoring systems are valuable tools for assessing FI and serve as a basis for discussion around the patient’s FI symptoms and when evaluating outcome after treatment/intervention.

The underlying etiology of FI in this study population varied, reflecting the variety of symptoms and etiologies presenting at the outpatient clinic in the everyday clinical setting. We consider this to be a strength, suggesting that LARS score should contribute with relevant information when assessing patients with FI in general. It would be interesting to further investigate the use of LARS score in subgroups with different etiologies to improve categorization [24].

As mentioned, Wexner score is the scoring system most frequently used by clinicians to assess FI. However, present data suggest that we need to expand the information provided. There are reports on the use of a combination of scoring systems when assessing FI, the most common being a combination of Wexner score [2] and quality-of-life assessment. Some have reported combining Wexner and St. Mark’s scores to assess FI [15].

Previous scoring systems have been criticized for not taking urgency and consumption of anti-diarrheal medication into consideration when assessing the severity of FI. In 1999, Vaizey et al. developed St. Mark’s score [25] which includes both urgency and use of anti-diarrheal medications. A weakness, however, was that instead of focusing on the frequency of urgency, the score was dichotomized into “yes” or “no” answers only. Since then, it has become obvious that clinicians must draw their own conclusions on how to interpret the question of urgency or not. As a result, definitions of urgency, registered as a maximum score of 4 points, varies [15], and criticism of St. Mark’s score not taking frequency of urgency into account is valid. The Memorial Sloan Kettering Bowel Function Instrument [26] could be an alternative complement to LARS score, though LARS score is preferable due to its clinically easy-to-use design [27]. LARS score is a well-known scoring system in both scientific and clinical fields and has been adopted by many clinics for the assessment of bowel dysfunction.

The LARS score for assessment of bowel dysfunction targets urgency a symptom typical of FI. LARS score has recently been employed to evaluate anorectal dysfunction among other patient groups such as after radical cystectomy, women suffering from deep endometriosis with bowel involvement, and patients with colon cancer [17–20]. This is because LARS score is a broad instrument for assessment of anorectal function and bowel dysfunction regardless of etiology. A combination of LARS and Wexner scores for assessment of long-term bowel dysfunction after low anterior resection has previously been suggested [28].

Normative data in the general population using LARS score show that approximately 10% of men and 19% of women between 50–79 years of age present with symptoms categorized as major LARS [21]. This suggests that the sensitivity of LARS score is high for assessment of bowel dysfunction in other patient groups than just those who have undergone low anterior resection for rectal cancer [29]. All symptoms should be considered when assessing FI, this includes urgency and fragmentation. LARS score is accepted and easy to use, and a combination of LARS and Wexner scores should improve assessment of FI.

The results of this study contribute to our current understanding regarding scoring systems for FI. There are many causes of FI, and it would be optimistic to believe that one scoring system alone could be applied to all cases of FI regardless of etiology. There are many underlying factors that must be considered, and the results of this study emphasize this point.

Spearman rank in the present study showed fair correlation only, except for the subgroup *injury located at IAS, EAS or both* where fair to moderate correlation was seen. The coefficients estimated here suggest that there is, at the most, only fair correlation between LARS and Wexner scores, with correlation coefficients using Spearman rank being somewhat higher than Kendall’s tau. It is known that Spearman rank correlation coefficients tend to be higher in medium to large samples compared to Kendall’s tau [30]. This probably explains the higher Spearman correlation coefficients seen in Table 2. Lack of correlation and agreement between the two scores shows that LARS score contribute with additional information to the Wexner score.

Correlation coefficients are reported differently depending on the field of research. In medical science, Chan et al. suggest Spearman rank correlation is *perfect, very strong, moderate, fair, poor,* and *zero* [31, 32].

There are questions in the LARS score that target urgency which is one of the most pronounced complaints among patients suffering from FI [29, 33]. That there was only slight correlation with Wexner score suggests that the scoring systems are likely to be complementary as was the hypothesis of this study.

The result in this study emphasizes the need for a more nuanced mapping when assessing FI. Usage of Wexner score as a single tool does not portray all the clinical manifestations of FI, e.g., urgency and fragmentation which are important entities to take in to consideration. Categorization of FI depending on presence of passive leakage or urgency has shown to be of clinical relevance [13, 14]. LARS score could, as suggested here, be used as a complementary tool for assessment of FI and could easily be incorporated into clinical practice. Clinicians have earlier provided information of combining different scoring systems in everyday practice when assessing FI [15]. Considering the results in this study, the authors recommend adding LARS score for assessment of FI in general. In a future perspective, it would be of interest to present data of LARS and Wexner scores depending on etiology of FI to further aid and guide the clinicians when assessing FI. This demands further research with a prospective approach. A prospective study should also be focusing management recommendations of FI depending on combination of the scores.

Limitation of this study was its retrospective design, and the number of participants would presumably have been higher if a prospective cohort had been chosen. As it was, a total of 27 patients were excluded. Since the aim was to study correlation and agreement between LARS and Wexner scores with no interfering factors, the number of subjects excluded was not significant. The cohort consists of a Swedish population in the northern part of Sweden from a single tertiary care center, a university hospital. Results in this study further emphasize the need for further research. Future studies should focus on a prospective approach and preferably include multiple centers.

## Conclusion

The LARS score will provide unique additional information to Wexner score when assessing FI and is not only suitable for use in assessing bowel dysfunction in patients who have been operated for rectal cancer. A combination of LARS score and Wexner score as a scoring system for assessment of FI should provide us with a more accurate picture of the symptomology of patients with FI regardless of etiology. This is of clinical relevance when making decisions on investigations, treatments, their use in general, and evaluation of treatment response, and will probably improve the situation of patients suffering from FI.

## Data Availability

The data that support the findings of this study are available upon reasonable request from the corresponding author. The data are not publicly available due to privacy or ethical restrictions
